# Supplementary material for: Unveiling the Bioactive Potential of the Invasive Jellyfish Phyllorhiza punctata Through Integrative Transcriptomic and Proteomic Analyses
Source: Biomolecules. 2025 Aug 4;15(8):1121. doi: 10.3390/biom15081121 (PMC12383608; doi:10.3390/biom15081121)
Supplement: Supplementary file 1 [file biomolecules-15-01121-s001.zip › Suplementary Material/FileS3/PEPFOLD4/XP_065057918_4421/clusters.html]

 PEP-FOLD clusters


Download archive of all models  here   

|  |  |  |
| --- | --- | --- |
| # | file | sOPEP |
| # cluster 1 - Size: 2 |  |  |
|  | XP\_065057918\_4421-model1 | -11.9113 |
|  | XP\_065057918\_4421-model1.1 | -11.9007 |
| # cluster 2 - Size: 4 |  |  |
|  | XP\_065057918\_4421-model2 | -11.6986 |
|  | XP\_065057918\_4421-model2.1 | -11.6857 |
|  | XP\_065057918\_4421-model2.2 | -11.6857 |
|  | XP\_065057918\_4421-model2.3 | -11.6054 |
| # cluster 3 - Size: 2 |  |  |
|  | XP\_065057918\_4421-model3 | -10.8278 |
|  | XP\_065057918\_4421-model3.1 | -10.7942 |
| # cluster 4 - Size: 2 |  |  |
|  | XP\_065057918\_4421-model4 | -9.69923 |
|  | XP\_065057918\_4421-model4.1 | -9.61047 |
| # cluster 5 - Size: 1 |  |  |
|  | XP\_065057918\_4421-model5 | -9.69505 |
| # cluster 6 - Size: 1 |  |  |
|  | XP\_065057918\_4421-model6 | -7.62106 |
| # cluster 7 - Size: 1 |  |  |
|  | XP\_065057918\_4421-model7 | -7.5944 |
| # cluster 8 - Size: 19 |  |  |
|  | XP\_065057918\_4421-model8 | -7.58515 |
|  | XP\_065057918\_4421-model8.1 | -7.31333 |
|  | XP\_065057918\_4421-model8.2 | -7.24684 |
|  | XP\_065057918\_4421-model8.3 | -7.22416 |
|  | XP\_065057918\_4421-model8.4 | -7.22414 |
|  | XP\_065057918\_4421-model8.5 | -7.21861 |
|  | XP\_065057918\_4421-model8.6 | -7.19282 |
|  | XP\_065057918\_4421-model8.7 | -7.15436 |
|  | XP\_065057918\_4421-model8.8 | -7.14432 |
|  | XP\_065057918\_4421-model8.9 | -7.12926 |
|  | XP\_065057918\_4421-model8.10 | -7.10569 |
|  | XP\_065057918\_4421-model8.11 | -7.05577 |
|  | XP\_065057918\_4421-model8.12 | -7.02425 |
|  | XP\_065057918\_4421-model8.13 | -7.02029 |
|  | XP\_065057918\_4421-model8.14 | -6.9835 |
|  | XP\_065057918\_4421-model8.15 | -6.9835 |
|  | XP\_065057918\_4421-model8.16 | -6.93878 |
|  | XP\_065057918\_4421-model8.17 | -6.93167 |
|  | XP\_065057918\_4421-model8.18 | -6.70353 |
| # cluster 9 - Size: 2 |  |  |
|  | XP\_065057918\_4421-model9 | -7.5516 |
|  | XP\_065057918\_4421-model9.1 | -7.39074 |
| # cluster 10 - Size: 4 |  |  |
|  | XP\_065057918\_4421-model10 | -7.51068 |
|  | XP\_065057918\_4421-model10.1 | -7.42542 |
|  | XP\_065057918\_4421-model10.2 | -7.14067 |
|  | XP\_065057918\_4421-model10.3 | -6.97415 |
| # cluster 11 - Size: 7 |  |  |
|  | XP\_065057918\_4421-model11 | -7.44249 |
|  | XP\_065057918\_4421-model11.1 | -7.33559 |
|  | XP\_065057918\_4421-model11.2 | -7.23946 |
|  | XP\_065057918\_4421-model11.3 | -7.14114 |
|  | XP\_065057918\_4421-model11.4 | -6.86034 |
|  | XP\_065057918\_4421-model11.5 | -6.73049 |
|  | XP\_065057918\_4421-model11.6 | -6.6988 |
| # cluster 12 - Size: 7 |  |  |
|  | XP\_065057918\_4421-model12 | -7.42444 |
|  | XP\_065057918\_4421-model12.1 | -7.42434 |
|  | XP\_065057918\_4421-model12.2 | -7.18156 |
|  | XP\_065057918\_4421-model12.3 | -7.12651 |
|  | XP\_065057918\_4421-model12.4 | -7.11279 |
|  | XP\_065057918\_4421-model12.5 | -6.94345 |
|  | XP\_065057918\_4421-model12.6 | -6.85137 |
| # cluster 13 - Size: 4 |  |  |
|  | XP\_065057918\_4421-model13 | -7.36585 |
|  | XP\_065057918\_4421-model13.1 | -7.09222 |
|  | XP\_065057918\_4421-model13.2 | -7.00873 |
|  | XP\_065057918\_4421-model13.3 | -6.89322 |
| # cluster 14 - Size: 3 |  |  |
|  | XP\_065057918\_4421-model14 | -7.35839 |
|  | XP\_065057918\_4421-model14.1 | -7.18346 |
|  | XP\_065057918\_4421-model14.2 | -7.17033 |
| # cluster 15 - Size: 7 |  |  |
|  | XP\_065057918\_4421-model15 | -7.3555 |
|  | XP\_065057918\_4421-model15.1 | -7.32663 |
|  | XP\_065057918\_4421-model15.2 | -7.29744 |
|  | XP\_065057918\_4421-model15.3 | -7.28063 |
|  | XP\_065057918\_4421-model15.4 | -7.16399 |
|  | XP\_065057918\_4421-model15.5 | -6.95553 |
|  | XP\_065057918\_4421-model15.6 | -6.78648 |
| # cluster 16 - Size: 9 |  |  |
|  | XP\_065057918\_4421-model16 | -7.29116 |
|  | XP\_065057918\_4421-model16.1 | -7.24204 |
|  | XP\_065057918\_4421-model16.2 | -7.1982 |
|  | XP\_065057918\_4421-model16.3 | -7.14016 |
|  | XP\_065057918\_4421-model16.4 | -7.08841 |
|  | XP\_065057918\_4421-model16.5 | -6.95139 |
|  | XP\_065057918\_4421-model16.6 | -6.82402 |
|  | XP\_065057918\_4421-model16.7 | -6.7524 |
|  | XP\_065057918\_4421-model16.8 | -6.66639 |
| # cluster 17 - Size: 1 |  |  |
|  | XP\_065057918\_4421-model17 | -7.20106 |
| # cluster 18 - Size: 2 |  |  |
|  | XP\_065057918\_4421-model18 | -7.18031 |
|  | XP\_065057918\_4421-model18.1 | -6.77937 |
| # cluster 19 - Size: 6 |  |  |
|  | XP\_065057918\_4421-model19 | -7.12186 |
|  | XP\_065057918\_4421-model19.1 | -7.11182 |
|  | XP\_065057918\_4421-model19.2 | -6.90389 |
|  | XP\_065057918\_4421-model19.3 | -6.88092 |
|  | XP\_065057918\_4421-model19.4 | -6.15502 |
|  | XP\_065057918\_4421-model19.5 | -6.14129 |
| # cluster 20 - Size: 3 |  |  |
|  | XP\_065057918\_4421-model20 | -6.99249 |
|  | XP\_065057918\_4421-model20.1 | -6.85 |
|  | XP\_065057918\_4421-model20.2 | -6.23407 |
| # cluster 21 - Size: 4 |  |  |
|  | XP\_065057918\_4421-model21 | -6.89057 |
|  | XP\_065057918\_4421-model21.1 | -6.84082 |
|  | XP\_065057918\_4421-model21.2 | -6.46133 |
|  | XP\_065057918\_4421-model21.3 | -5.29897 |
| # cluster 22 - Size: 3 |  |  |
|  | XP\_065057918\_4421-model22 | -5.14034 |
|  | XP\_065057918\_4421-model22.1 | -4.61759 |
|  | XP\_065057918\_4421-model22.2 | -3.46977 |
| # cluster 23 - Size: 2 |  |  |
|  | XP\_065057918\_4421-model23 | -4.67395 |
|  | XP\_065057918\_4421-model23.1 | -3.93414 |
| # cluster 24 - Size: 2 |  |  |
|  | XP\_065057918\_4421-model24 | -4.64805 |
|  | XP\_065057918\_4421-model24.1 | -4.53117 |
| # cluster 25 - Size: 1 |  |  |
|  | XP\_065057918\_4421-model25 | -4.53804 |
| # cluster 26 - Size: 1 |  |  |
|  | XP\_065057918\_4421-model26 | -4.36721 |
|  |  |  |
